# Young, Healthy Subjects Can Reduce the Activity of Calf Muscles When Provided with EMG Biofeedback in Upright Stance

**DOI:** 10.3389/fphys.2016.00158

**Published:** 2016-04-29

**Authors:** Taian M. Vieira, Stéphane Baudry, Alberto Botter

**Affiliations:** ^1^Laboratorio di Ingegneria del Sistema Neuromuscolare, Dipartimento di Elettronica e Telecomunicazioni, Politecnico di TorinoTorino, Italia; ^2^Escola de Educação Física e Desportos, Departamento de Arte Corporal, Universidade Federal do Rio de JaneiroRio de Janeiro, Brasil; ^3^Laboratory of Applied Biology and Neurophysiology, ULB Neuroscience Institute, Université libre de BruxellesBrussels, Belgium

**Keywords:** balance, standing, biofeedback, electromyography, postural sway

## Abstract

Recent evidence suggests the minimization of muscular effort rather than of the size of bodily sway may be the primary, nervous system goal when regulating the human, standing posture. Different programs have been proposed for balance training; none however has been focused on the activation of postural muscles during standing. In this study we investigated the possibility of minimizing the activation of the calf muscles during standing through biofeedback. By providing subjects with an audio signal that varied in amplitude and frequency with the amplitude of surface electromyograms (EMG) recorded from different regions of the gastrocnemius and soleus muscles, we expected them to be able to minimize the level of muscle activation during standing without increasing the excursion of the center of pressure (CoP). CoP data and surface EMG from gastrocnemii, soleus and tibialis anterior muscles were obtained from 10 healthy participants while standing at ease and while standing with EMG biofeedback. Four sensitivities were used to test subjects' responsiveness to the EMG biofeedback. Compared with standing at ease, the two most sensitive feedback conditions induced a decrease in plantar flexor activity (~15%; *P* < 0.05) and an increase in tibialis anterior EMG (~10%; *P* < 0.05). Furthermore, CoP mean position significantly shifted backward (~30 mm). In contrast, the use of less sensitive EMG biofeedback resulted in a significant decrease in EMG activity of ankle plantar flexors with a marginal increase in TA activity compared with standing at ease. These changes were not accompanied by greater CoP displacements or significant changes in mean CoP position. Key results revealed subjects were able to keep standing stability while reducing the activity of gastrocnemius and soleus without loading their tibialis anterior muscle when standing with EMG biofeedback. These results may therefore posit the basis for the development of training protocols aimed at assisting subjects in more efficiently controlling leg muscle activity during standing.

## Introduction

During upright standing, the human body oscillates continuously and spontaneously. Three key mechanisms collectively contribute to the control of the standing posture: (i) the passive ankle stiffness, (Loram and Lakie, 2002); (ii) sensory information integrated at spinal and supraspinal levels (Peterka, 2002; Kiemel et al., 2011) and; (iii) activation of muscles involved in postural control (Bottaro et al., 2008; Vieira et al., 2012; Elias et al., 2014). When the integrity of any of these intrinsic sources of control is compromised (e.g., with aging, Horak et al., 1992; Inglis et al., 1994), the amplitude of body sways increases and balance is threatened. Several types of intervention were proposed in the literature to recover and/or preserve these mechanisms within functional levels. Among these interventions, exercises targeting the stimulation of sensory-motor pathways relevant for posture control (e.g., TAI-Chi-Chuan and slack-line training, Li et al., 2005; Keller et al., 2012; Penzer et al., 2015) have mainly been investigated.

Recent innovative approaches to improve balance control consist of using feedback of balance performance. The position of the body center of pressure (CoP) and trunk acceleration, both in medio-lateral and anterior-posterior directions, have been considered as key feedback variables in the literature (Chiari et al., 2005; Ledebt et al., 2005; Sayenko et al., 2010; Mirelman et al., 2011). More specifically, subjects are expected to minimize their postural sways in response to auditory and visual clues reflecting their CoP position or trunk acceleration. Compared with conventional training programs (balance or strength), biofeedback has the advantage of providing subjects with postural information rarely perceived (e.g., the bodily sways). A corollary of the use of feedback systems for balance training is the assumption that minimization of postural sways reflects an improvement in balance control (Dozza et al., 2005; Ledebt et al., 2005; Sayenko et al., 2010).

An alternative feedback approach for balance training may rely on the use of biological-related signals, such as electromyograms (EMGs). According to recent evidence, efficient control of posture is more likely associated with minimization of unnecessary muscle activity rather than of postural sways; muscles may not be activated continuously to control posture (Vieira et al., 2012) and the degree of activation may be scaled with sway size (Sakanaka et al., 2016). EMGs and skeletal muscle ultrasonography, indeed, revealed timely, intermittent modulations in calf muscles' activity during standing, with alternate periods of muscle activity and silencing (Loram et al., 2005; Vieira et al., 2012; Héroux et al., 2014). As suggested by Loram et al. (2009), differently from a continuous control of muscle activation, such intermittency provides the nervous system with a time window over which sensory feedback from muscle, joint and tendon receptors is not affected by the efferent drive to skeletal muscles. Moreover, theoretical predictions indicate less energy is spent by a postural controller relying on intermittent rather than on continuous control mechanisms (Bottaro et al., 2008). Most importantly, in the view of an intermittent control paradigm, the body sways may be attenuated though not suppressed. Indeed, the size of sways and the amount of muscle activity seem to be scaled inversely during standing; minimization of sways demands high muscle effort and minimization of muscle effort leads to large, postural sways (Kiemel et al., 2011). It is therefore possible that EMG-based feedback results in a more efficient control of standing.

This study addresses the question of whether the activation of ankle muscles may be minimized during upright standing through EMG biofeedback. By hearing a sinusoidal audio signal, modulated according to EMGs recorded from the calf muscles, subjects are expected to reduce the activity of plantar flexor muscles to quiet standing levels. Given that minimization of muscle activity may result in excessively large postural sways (Kiemel et al., 2011), and thus threaten stability, we further investigate whether reducing muscle activity is associated with altered CoP displacements. To our knowledge this is the first report showing the potentialities of EMG-audio biofeedback for the balance training.

## Methods

### Participants

Ten male subjects (range values: age 22–38 years; body mass 65–87 kg; height 165–187 cm) were recruited to participate in this study. None of them reported any neuromuscular disorders that could affect standing, both prior to and in occasion of the experiments. All participants were instructed in relation to the experimental protocol before providing written, informed consent. The experimental procedures conformed to the latest revision of the Declaration of Helsinki and were approved by the Ethics Committee of Politecnico di Torino.

### Experimental protocol

All participants performed stabilometric tests on a force-plate, lasting 40 s each. Subjects were instructed to stand with both feet at a comfortable position, arms alongside the body and eyes open. An audio stimulus proportional to the level of calf muscle activity was provided to participants through headphones for 40 s and they were asked to minimize it. As no evidence was found on the appropriate coding for EMG-audio feedback during standing, and given that subjects may be not able to control a very sensitive EMG-based audio stimulus (i.e., high gain possibly limits the ability to finely adjust EMG level), four different sensitivities were used to code the volume and the frequency of the audio signal from the level of muscle activity. A total of five conditions were applied, one without and four with EMG-audio feedback. When not provided with audio feedback, participants were engaged into active conversation to suppress the conscious control of postural sways (Loram et al., 2001). This condition was regarded as a reference, standing condition, hereafter termed *standing at ease*. During the EMG-biofeedback conditions, the subjects were not engaged in conversation. For the *standing at ease* and the four EMG-biofeedback conditions, subjects were not allowed to move their head, trunk and upper and lower limbs. Trials started over in the case any gross movements were perceived by the investigators. Two trials were applied randomly for each standing condition, in random order, and at least 5 min of rest was provided between trials. Data recording started after subjects felt acquainted with the feedback tasks; familiarization periods lasted a few minutes.

Even though participants were free to select their preferred feet position, they were not allowed to change the support base across the stabilometric trials. Marks on the force-plate, drawn in correspondence of the tip of the talus and of the head of the first and third metatarsal bones, ensured participants kept the same foot position during all standing conditions. Stance width, defined as the distance between midpoints from the talus to the head of the third metatarsal, ranged from 21.3 to 24.5 cm across participants. Given the active torque at ankle and hip joints has been shown to scale with stance width (Bingham and Ting, 2013), we did not allow subjects to change their stance width across the five balance conditions. Otherwise, EMG-audio feedback and stance width would both contribute to differences in the degree of muscle activity across the standing conditions.

### Positioning electrodes and recording EMGs and ground reaction forces

Fifteen single-differential EMGs were detected from the tibialis anterior and the medial and lateral gastrocnemius muscles in the right leg with three arrays of surface electrodes, one for each muscle (16 silver bar electrodes; 10 × 1 mm; 10 mm inter-electrode distance; SpesMedica, Battipaglia, Italy). These arrays were positioned parallel to the longitudinal axis of the three muscles. For the gastrocnemius heads, the most proximal electrode of each array was positioned on average 2.1 cm distant from the popliteal crease (*N* = 10 subjects). Such positioning ensured the action potentials of fibers residing in different proximo-distal gastrocnemius regions to be represented in the surface EMGs (Vieira et al., 2011). For the tibialis anterior muscle, the linear array was positioned parallel and just lateral to the anterior crest of the tibia, with its center coinciding with the bulk of the muscle. From the soleus muscle, single-differential EMGs were detected with two smaller arrays (8 silver bar electrodes; 10 × 1 mm; 5 mm inter-electrode distance). Arrays were positioned with their center located 3 cm distally from the junction between the Achilles tendon and the medial and lateral gastrocnemius heads. The arrays of electrodes were used to provide representative EMGs of the soleus lateral and medial aspects (Reffad et al., 2014). Electrodes were positioned after abrading and cleansing the skin with abrasive paste and alcohol.

The amplification factor ranged from 1000 to 5000 to ensure no saturation and to maximize the signal to noise ratio, (15–750 Hz bandwidth; EMG-USB amplifier; LISiN and OT Bioelettronica, Turin, Italy). EMGs and the ground reaction forces measured with a piezoelectric force-plate (9286AA Kistler, Milan, Italy) were sampled synchronously at 2048 Hz with a 12 bits A/D converter. The surface EMGs and the center of pressure (CoP) position, calculated from the ground reaction forces, were stored for further analysis.

### Modulating audio signals from surface EMGs

**Figure 1:**
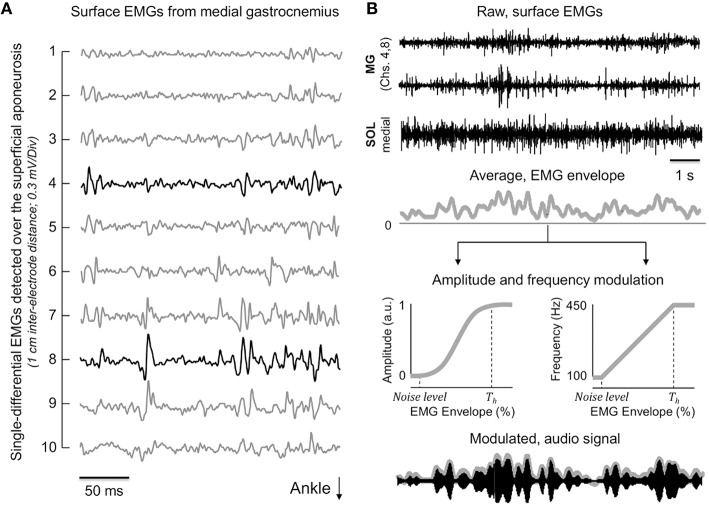
**Coding audio-feedback from surface EMGs. (A)** Shows a short epoch of raw, single differential EMGs detected from the medial gastrocnemius muscle. Channels (i.e., pairs of electrodes) from 1 to 10 were located at skin regions covering the gastrocnemius superficial aponeurosis. Black traces indicate EMGs considered for modulating the audio signal (top two traces in **B**). **(B)** Provides a schematic representation of how the sinusoidal, audio signal was obtained from EMGs. Note the correspondence between fluctuations in the amplitude of the EMG envelope averaged across traces shown in **(B)** and the modulated sinusoid (bottom traces). The amplitude and the frequency of this sinusoid were respectively modulated according to a sigmoid and linear function. These functions were defined based on a threshold (*T*_*h*_), set in relation to the amplitude distribution of the EMG envelope obtained during 20 s of quiet standing (see text).

Initially, EMGs were visually inspected to identify the channels detecting action potentials from different motor units (Figure 1A). Considering surface EMGs detected from a single muscle region may not represent the whole muscle activity (Vieira et al., 2011), EMGs collected from different regions within the same muscle were considered for the feedback, audio modulation; one or two channels were selected for the soleus muscle, depending on whether action potentials of different motor units could be observed in different channels or not, whereas two to three channels were used for the medial gastrocnemius. As no modulation was observed in EMG amplitude for the lateral gastrocnemius head and tibialis anterior during preliminary experiments, channels from these muscles were not considered for the audio feedback. The EMGs detected by the channels selected were full-wave rectified and smoothed with a low-pass, 4th order Butterworth filter (3 Hz cut-off frequency; Héroux et al., 2014). These smoothed EMGs (i.e., EMG envelopes) were averaged across channels and muscles to provide an EMG envelope representing the activity of different motor units within and between plantar flexors (Figure 1B). The amplitude of such averaged envelope, i.e., the level of plantar flexors activity, was finally used to code the audio, feedback signal.

The amplitude and the frequency of the audio signal were coded differently from EMGs (Figure 1B). A sigmoid function was considered to modulate the sinusoid amplitude (Figure 1B). On the other hand, changes in the sinusoid frequency were linearly related to changes in the amplitude of the average, EMG envelopes. This coding has been used previously to provide subjects with audio feedback from trunk accelerations during standing (Chiari et al., 2005). In relation to the linear modulation, previous evidence has shown the CoP-audio feedback coded with a sigmoid function leads to better performance during standing (Dozza et al., 2006).

Before modulating the audio stimulus from muscle activity, EMG envelopes were normalized with respect to a reference, standing condition: subjects were asked to sway as much as possible back and forward. They were specifically instructed to lean their body exclusively around the ankle joint, without lifting up their fore and rear foot and at their preferred speed. A total of five consecutive sways were recorded. From this calibration data, the EMG envelope was computed as indicated above. The 5th and the 95th percentiles of the EMG envelope obtained during the voluntary sways were respectively used for the subtraction of the background noise and for the amplitude normalization of the EMG envelope computed when standing with EMG biofeedback.

**Figure 2:**
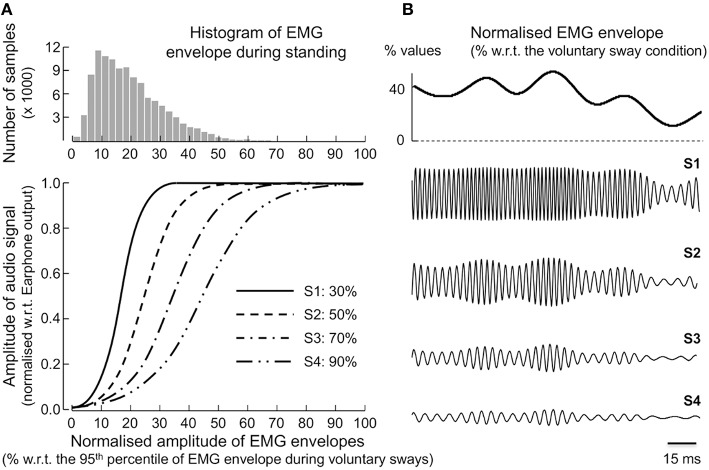
**Different sensitivities for EMG-biofeedback modulation. (A)** Shows the four sigmoid functions (bottom panel) considered to modulate the amplitude of the sinusoidal, audio signal. Each sigmoid was created from thresholds (*T*_*h*_) set according to the 95th percentile (i.e., 100% in the *x* axis) of the EMG envelope obtained during the voluntary sways condition: S1 (*T*_*h*_ = 30%); S2 (*T*_*h*_ = 50%); S3 (*T*_*h*_ = 70%); S4 (*T*_*h*_ = 90%). The histogram shown in the top panel was created from the EMG envelope obtained in preliminary experiments (single participant; 20 s of *standing at ease*; averaged across soleus and medial gastrocnemius muscles) sought to define appropriate *T*_*h*_ values for the testing of EMG-biofeedback sensitivity. Examples of sinusoids modulated from amplitude of the mean EMG envelope are shown in **(B)**, for each of the sensitivities tested (i.e., for each threshold selected).

Different sensitivities were considered to modulate the audio stimulus. These sensitivities were obtained by changing the steepness of the sigmoid and linear functions in relation to the EMG amplitude. The amplitude *a*[*n*] of the audio signal was modulated as:

(1) $$\begin{array}{rcl} a\left[n\right] & = & \frac{1}{\left(1+e^{-c\left(EMG\left[n\right]-b\right)}\right)} \end{array}$$

(2) $$\begin{array}{rcl} b & = & \frac{T_{h}}{2} \end{array}$$

(3) $$\begin{array}{rcl} c & = & \frac{4.6}{b} \end{array}$$

where *EMG*[*n*] corresponds to the samples of EMG envelopes, after background noise subtraction and normalization, and *T*_*h*_ stands for the threshold defining the sensitivity of the EMG-audio modulation. For example, when the EMG envelope reaches *T*_*h*_ the intensity of the audio stimulus reaches 99% of its maximal value. Four different EMG-biofeedback sensitivities were then defined by setting *T*_*h*_ to 30% (S1), 50% (S2), 70% (S3), and 90% (S4) of the 95th percentile of the distribution of EMG envelope obtained during the calibration phase: preliminary experiments revealed subjects were not able to respond to the feedback stimulus by setting the threshold to less than 30% as such a small threshold typically easily resulted in saturation of the audio stimulus during *standing at ease* (Figure 2). Figure 2B shows examples of a short epoch of sinusoids modulated according to the different sensitivities tested.

The frequency *f* [*n*] of the sinusoidal, audio signal was modulated according to:

(4) $$\begin{array}{l} f[n]=\{\begin{array}{cc} EMG[n]\cdot m+100, & EMG[n]<T_{h}\\ 450, & EMG[n]\geq T_{h} \end{array} \end{array}$$

with the angular coefficient *m* being defined as:

(5) $$\begin{array}{l} m=\frac{350}{T_{h}} \end{array}$$

EMG biofeedback was provided to participants in real time. A custom Matlab script (MathWorks Inc., MA, USA) was written to output the modulated, audio signal into the soundcard of a personal computer. The audio stimulus was then transmitted wirelessly to headphones worn by participants. The time taken to compute and average envelopes and modulate the sinusoid ranged from 10 to 20 ms. The modulated sinusoid was then buffered and sent to the soundcard at a fixed, 50 ms interval; the same refresh rate considered by Chiari et al. (2005).

### Quantifying the effect of EMG biofeedback during standing

Variations in the degree of calf muscle activity and in CoP position were calculated through the root mean square (RMS) amplitude. The RMS values were calculated from band-pass filtered EMGs (4th order, bidirectional Butterworth filter, 20–350 Hz cut-off frequencies) and then averaged separately for each muscle, over all channels in the array and over the whole standing test (40 s). In agreement with previous accounts reporting the short, modal duration of individual body sways (less than 2 s; Bottaro et al., 2008; Vieira et al., 2012; Héroux et al., 2014), RMS values for the CoP position in the sagittal plane were computed over short epochs of 2 s; these RMS values reflect the changes in CoP position occurring within individual bodily sways rather than changes in the mean CoP position (Loram et al., 2001; Vieira et al., 2010).

### Statistics

As data distributions could be fitted by a Gaussian function (Shapiro-Wilk statistics; *P* > 0.15 for all variables), parametric statistics were used to quantify the effect of EMG biofeedback on the minimization of muscle activity and on the size of CoP sways. The RMS amplitude of surface EMGs, obtained during *standing at ease* and EMG-biofeedback conditions, was compared using one-way ANOVA, with the five standing conditions as repeated measures (without feedback and with the four audio-feedback sensitivities, Figure 2). The same analysis was considered to compare the mean CoP position and the size of CoP sways between standing conditions, both in the frontal and sagittal planes. Whenever an additive effect was observed, paired comparisons were assessed with the Newman–Keuls *post-hoc* analysis. Data was presented as mean and standard deviation.

## Results

### EMG activity during standing

**Figure 3:**
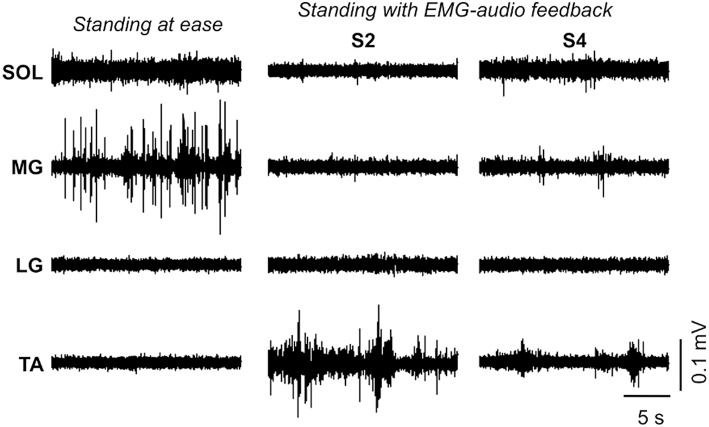
**Effect of EMG- biofeedback on the EMG amplitude profile**. Raw surface EMGs are shown for a single participant during *standing at ease* (left column) and when standing with EMG-biofeedback, for the greatest (middle column; S2) and the lowest (right column; S4) sensitivities. To better illustrate the differences in EMG amplitude between conditions, only a single signal is shown per muscle. See Figure 2 for specific indications on the sensitivity of EMG-audio coding. Signals are shown over a relatively short epoch (20 s) to facilitate observing the modulations in EMG amplitude for the different standing conditions. EMGs from different muscles are shown in different rows, from top to bottom: soleus medial region; medial gastrocnemius; lateral gastrocnemius; tibialis anterior.

Marked differences were observed in the amplitude and in the profile of surface EMGs detected during the different standing conditions. As illustrated for a single participant in Figure 3, ankle plantar flexors showed EMGs with markedly greater RMS amplitude during *standing at ease* than during standing with biofeedback, regardless of the EMG-audio sensitivity considered. Even though the EMG amplitude profile differed between the medial head (intermittent) and the lateral gastrocnemius head (no modulation) and soleus muscle (continuous) during *standing at ease*, all plantar flexors showed diminished RMS values when standing with EMG biofeedback (occasional bursts for gastrocnemius and smaller baseline amplitude for soleus; cf. columns in Figure 3). The opposite was observed for the ankle dorsal flexor. Lower EMGs were observed for the tibialis anterior muscle during *standing at ease* than during standing with EMG biofeedback, in particular for the most sensitive feedback (cf. S2 in Figure 3). For the least sensitive EMG biofeedback, however, increases in EMG amplitude for the tibialis anterior occurred rarely and lasted shortly (cf. S4 in Figure 3).

**Figure 4:**
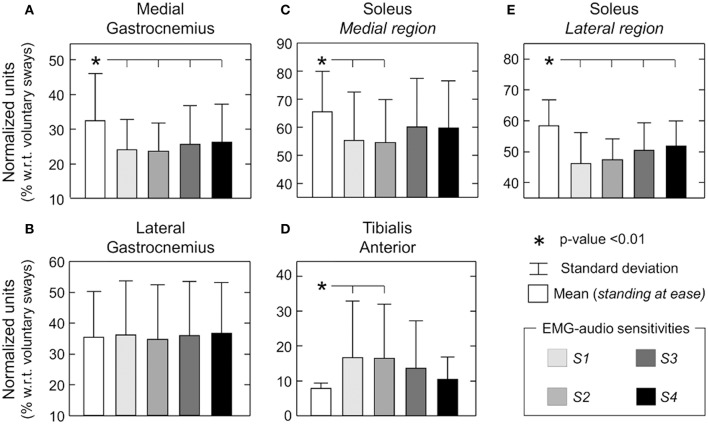
**EMG biofeedback on the minimization of EMG amplitude**. Mean and standard deviation (whisker) values are shown for the RMS amplitude of surface EMGs collected during different standing conditions, from standing without feedback (white bar) to the least sensitive (S4; Figure 2) EMG-biofeedback condition (black bar). Values are shown for each of the five muscles studied: **(A)** medial gastrocnemius; **(B)** lateral gastrocnemius; **(C)** soleus medial portion; **(D)** tibialis anterior; **(E)** soleus lateral portion. Asterisk denotes statistical significance (*P* < 0.05).

Group data revealed the effect of EMG biofeedback on EMG amplitude varied significantly with muscle and feedback sensitivity (ANOVA main effect; *P* < 0.05 for tibialis anterior, medial gastrocnemius and soleus muscles). The RMS amplitude of EMGs detected from the medial gastrocnemius and from the lateral aspect of the soleus muscle was significantly lower (10–15%) when standing with EMG biofeedback than standing naturally, for the four sensitivities tested (Figures 4A,E; Newman-Keuls *post-hoc*; *P* < 0.05 in all cases; *N* = 50: 10 subjects × 5 standing conditions). For the soleus medial portion, when compared with *standing at ease*, significantly smaller RMS values were obtained only for the S1 and S2 EMG-audio sensitivities (Figure 4C; Newman-Keuls *post-hoc*; S1: *P* = 0.001; S2: *P* = 0.001; S3: *P* = 0.084; S4: *P* = 0.063). In contrast, EMG biofeedback did not influence the RMS values for the lateral gastrocnemius (Figure 4B; ANOVA additive effect; *P* = 0.44). Finally, when compared to the RMS values obtained during *standing at ease*, the RMS amplitude obtained during feedback for the tibialis anterior muscle were statistically larger for the two most sensitive conditions (Figure 4D; Newman-Keuls *post-hoc*; S1: *P* = 0.012; S2: *P* = 0.014). For the two least sensitive conditions, EMGs collected from tibialis anterior were however not greater than those detected during the reference, *standing at ease* condition (S3: *P* = 0.089; S4: *P* = 0.428). When specifically considering the least sensitive condition, results in Figure 4 indicate the amplitude of EMGs detected from ankle plantar flexors decreased by ~7% whereas the RMS values of the ankle dorsi-flexor increased by ~2% (cf. mean RMS values between standing at ease and S4 for medial gastrocnemius, medial and lateral soleus and tibialis anterior muscles).

### Changes in CoP position and displacements

**Figure 5:**
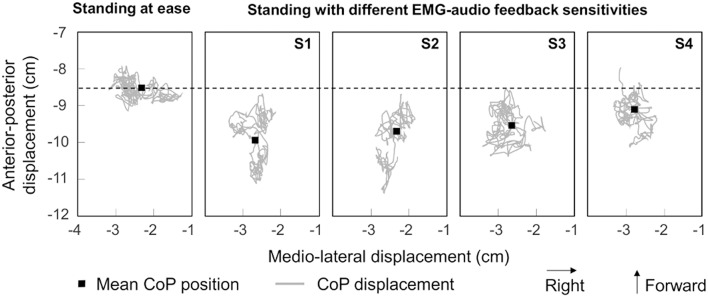
**Variation in the distribution of CoP position with EMG-biofeedback**. The CoP position (gray traces) calculated during the whole registration (40 s) is shown for a representative participant, during *standing at ease* (left) and during the four feedback standing conditions, from the most sensitive (S1) to the least sensitive (S4) condition (cf. Figure 2). The coordinates of the mean position of the feet CoP (black square) in the anterior-posterior and medio-lateral directions are shown during *standing at ease* and during EMG-biofeedback conditions.

EMG biofeedback influenced to a greater extent CoP position and displacements in the sagittal than in the frontal plane. Data from a single, representative participant indicate that, for the five standing conditions, the CoP occupied on average the same medio-lateral region on the force-plate (Figure 5). In the sagittal plane, conversely, the mean CoP position was localized at more posterior regions with than without EMG biofeedback. Even though feet position on the force-plate did not change between conditions, the mean CoP position was localized ~3 and ~1 cm posterior to the mean CoP position obtained during *standing at ease* for the most and the least sensitive EMG-audio coding, respectively (cf. the relative position of black squares in Figure 5). Close inspection of Figure 5 further reveals the CoP spanned a progressively smaller anterior-posterior region as sensitivity decreased.

**Figure 6:**
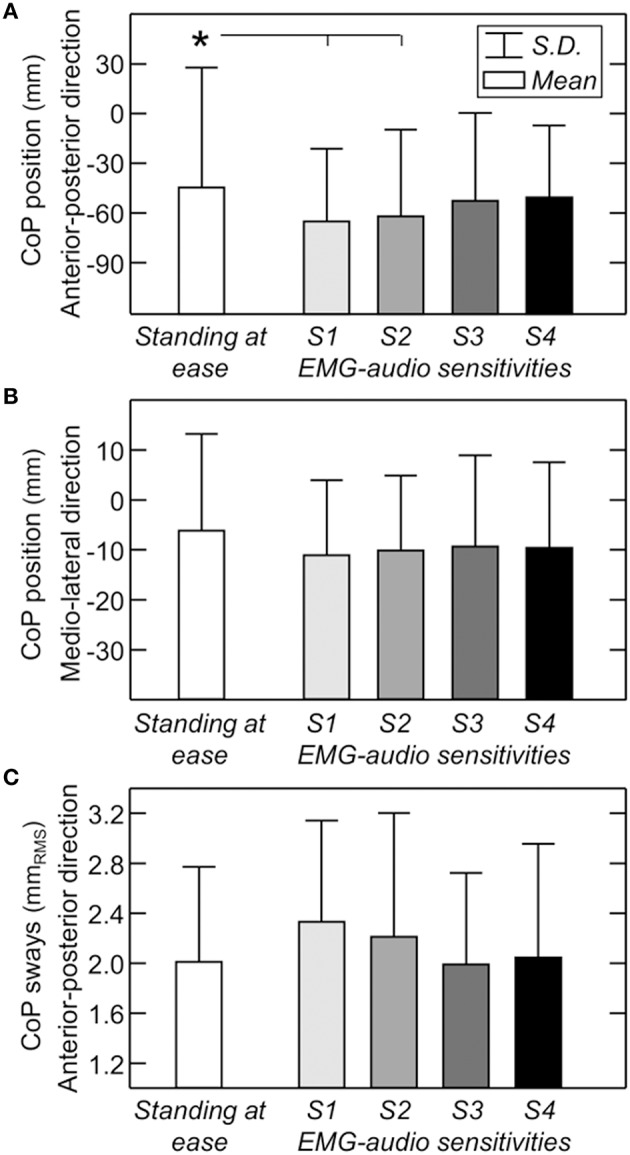
**EMG-biofeedback effect on the postural sways**. Mean and standard deviation (whisker) values are shown for the mean CoP position along the anterio-posterior **(A)** and medio-lateral **(B)** directions and for the RMS amplitude of CoP position calculated over epochs of 2 s **(C)**. Values are shown for the different standing conditions, from standing without feedback (white bar) to the least sensitive (S4; Figure 2) EMG-audio biofeedback condition (black bar). Asterisk denotes statistical significance (*P* < 0.05).

When considering all participants, a significant effect of EMG biofeedback was exclusively observed for the CoP mean position in the sagittal plane. For the two most sensitive conditions, CoP position was localized at significantly more posterior regions when compared to the *standing at ease* condition (Figure 6A; Newman-Keuls *post-hoc*; S1: *P* = 0.022; S2: *P* = 0.050; S3: *P* = 0.355; S4: *P* = 0.110). In the medio-lateral direction, the CoP position did not change with the standing condition (Figure 6B; ANOVA additive effect; *P* = 0.094). Even though the RMS amplitude of CoP displacements computed for the most sensitive feedback condition was slightly larger (0.4 mm) than that obtained during *standing at ease*, no significant effect of EMG biofeedback on the size of postural sways was observed for any of the four sensitivities considered (Figure 6C; ANOVA additive effect; *P* = 0.133).

## Discussion

In this study we investigated whether subjects can minimize their calf muscles' activity during standing when provided with EMG biofeedback. Different sensitivities of EMG biofeedback were tested, with the less sensitive conditions (S3 and S4) leading to a better compromise between changes in EMG amplitude and CoP parameters (Figures 4, 6). Specifically for the S3 and S4 conditions, when compared to *standing at ease*, key results revealed: (i) the amplitude of EMGs collected from medial gastrocnemius and soleus muscles decreased significantly; (ii) tibialis anterior (i.e., antagonist) activity increased marginally; (iii) CoP displacement and mean position did not change significantly. These results suggest EMG biofeedback may assist subjects in reducing the activity of calf muscles without altering the size of CoP sways.

### Methodological considerations

Different modalities could have been considered for the presentation of EMG biofeedback to participants. Typically, EMG biofeedback is provided either through auditory or visual signal, with auditory (e.g., pitch tone, pulse frequency) or visual (e.g., needle meter, LED bar) parameters varying with the firing rate of individual motor units or with the amplitude of EMGs (Basmajian, 1963; Poppen et al., 1988; Park and Yoo, 2012). Visual cues associated with the surroundings is however critical for the control of posture, in particular when the vestibular or proprioceptive feedback sources are not accurate (Bugnariu and Fung, 2007). For this reason, auditory feedback seems more appropriate than visual feedback for the repression of muscle activity in standing; the different sensory pathways ensure EMG feedback and the visual information do not dispute for internal, processing resources.

During standing, postural sways change with an assortment of factors. Of particular relevance for the interpretation of current findings is the effect of articulation, that is, of the postural perturbations induced by spoken tasks. Yardley et al. (1999), for example, observed significant increases in postural sways when subjects stood upright while repeating numbers aloud. Notwithstanding this articulation effect, in the *standing at ease* condition, we decided to engage subjects in meaningful conversation than to instruct them to stand quietly. Two reasons motivated our decision. First, the increased postural sways observed by Yardley et al. (1999) do not necessarily mean increased muscle activity. Loram et al. (2001), for example, observed the size of postural sways rather than the level of muscle activation increases during standing while talking. Second, and most importantly, by engaging subjects in conversation we expected all subjects to be in a similar, reference condition. Otherwise, leaving subjects uninstructed about the task or instructing them to stand still could lead to inter-individual differences in the degree of attention and thus to marked differences in muscle activation during *standing at ease* between subjects. Moreover, cognitive effects on the level of muscle activation are not so well documented as on CoP and postural sways during standing (Stoffregen et al., 2000; Nafati and Vuillerme, 2011); recent evidence suggests however the cognitive task difficulty does not affect EMG amplitude (Baudry and Gaillard, 2014). We therefore presume *standing at ease* condition posits a physiologically relevant, reference condition for which responses to EMG biofeedback were compared.

### EMG biofeedback minimizing muscle activation during standing

Of crucial importance for EMG-audio feedback is ensuring subjects are provided with feedback information that increases their awareness on a physiological parameter of interest—the neural drive to plantar flexors in the present study—that they can modulate (Basmajian, 1963). On this view, the audio feedback should respond to variations in EMG activity of the calf muscles exclusively associated with compensation of postural sways during standing. For very sensitive EMG biofeedback (S1 and S2), however, small changes in EMG amplitude suddenly increase the audio level toward saturation (see Supplementary Material). Considering the variations in the amplitude of calf muscles' EMGs during standing are small (up to 50 μV_pp_ for gastrocnemius; Joseph et al., 1955), the very sensitive feedback audio likely unduly emphasized the variations in muscle activity unrelated to sway control (e.g., due to electronics and synaptic noise) while being not sensitive to the modulations in muscle activity associated with compensation of postural sways, in particular for large sways (i.e., as audio volume and frequency easily saturated; Figure 2). Consequently, all subjects stated standing in S1 and S2 conditions was unpleasant because of the resulting sound and the difficulty to adjust it. Elimination of these spurious sources of variation in EMG amplitude would demand abolishing the neural drive to plantar flexors, which would imply moving the body posteriorly at the potential cost of eliciting activation of ankle flexors. In agreement, the most sensitive coding was accompanied by significant posterior shifts of body CoP, increased CoP displacements, decreased plantar flexors activity and increased tibialis anterior activity in relation to *standing at ease* (Figures 3–6). According to the aims of this study, these results seem to discourage considerations on the use of highly sensitive (S1 and S2 conditions) EMG-audio biofeedback for the general relaxation of postural muscles.

Participants performed generally better in terms of controlling their muscle activation when standing during the least sensitive EMG-biofeedback conditions. Given that during S3 and S4 conditions the audio signal was not as sensitive to slight increases in EMG amplitude from background level as that provided by S1 and S2 conditions, none of the subjects reported the audio stimulus to be unpleasant. Furthermore, S3 and S4 did not lead to saturation in audio tone and pitch; these sensitivities provided subjects with an audio stimulus whose amplitude and frequency responded over a wider range of EMG amplitude in relation to the S1 and S2 conditions (Figure 2). Under S3 and S4 conditions, participants significantly reduced the general level of plantar flexors activity, did not increase the level of dorsal flexor activation and kept the mean CoP position and the amplitude of CoP sways within the *standing at ease* levels.

### Minimization of the activity of postural muscles through EMG biofeedback

EMG biofeedback has found relevant application for neuromuscular re-education; i.e., for mitigating the coordination of muscles' activity necessary to accomplish a specific motor, cognitive or behavioral goal. Such re-education may be achieved by training subjects in repressing abnormal muscle activity (Wright et al., 2013) or in eliciting dormant muscles (Franz et al., 2014). In the present study, EMG biofeedback was used to investigate whether subjects are capable of repressing muscle activity during standing. The potential benefits of EMG biofeedback for postural control are discussed in the next subsection whereas here we discuss how subjects may have succeeded in minimizing their postural activity.

During *standing at ease*, the vertical projection of the body center of mass is, on average, localized anteriorly to the ankle joint. As a consequence, postural sways are regulated mainly through activation of ankle plantar rather than dorsal flexors. Moving the body center of mass toward the ankle joint (i.e., posteriorly) is therefore one potential mechanism to suppress activation of ankle extensors. This possibility seems unlikely as the mean CoP position and the amplitude of CoP displacements in the anterior-posterior axis did not change significantly from *standing at ease* to standing with the least sensitive EMG biofeedback (S3 and S4 in Figures 6A,C). Unloading the right leg (i.e., where surface electrodes were positioned) posits an alternative explanation for the feedback-induced relaxation of ankle extensors. This possibility is similarly discredited as CoP mean position in the lateral axis did not change significantly with standing condition (Figure 6B), suggesting the amount of loading between legs (Genthon et al., 2008) did not change from *standing at ease* to standing with biofeedback. It is however possible that the combined, non-significant changes in CoP position in both axes may have contributed to reduce the EMG of ankle plantar flexors without consistently eliciting the tibialis anterior muscle during the least sensitive feedback conditions (S3 and S4 in Figure 4). Verification of this possibility is currently unviable as no evidence was found on how the amplitude of EMGs from each ankle plantar flexor changes with CoP position in the anterior-posterior and medio-lateral axes. It is also possible that the EMG biofeedback has stimulated intrinsic mechanisms responsible for the diminished neural drive and thus activation of ankle muscles. The degree of involvement of central and peripheral resources in calf muscles' relaxation is not predictable, in particular because both central processing and spinal circuitry have been suggested to mediate the corrective responses to postural sways during standing (Loram et al., 2009; Elias et al., 2014). Even though further work is necessary to better understand the mechanisms involved in the modulation of muscle activity with biofeedback, according to our results participants were able to reduce the activity of ankle extensors and flexors through EMG biofeedback.

### Potential benefits of EMG biofeedback for the control of the standing posture

The feedback source considered in the present study is different from the feedback sources typically used for postural training. While audio-visual stimuli based on body kinematics and kinetics constitute an additional source to the sensorial feedback mediated by proprioceptive, vestibular and visual systems, potentially useful for teaching subjects to decrease or maintain the amplitude of their postural sways (Chiari et al., 2005; Ledebt et al., 2005; Sayenko et al., 2010; Mirelman et al., 2011), EMG feedback provides subjects with sensory information otherwise not available to them. Although the degree of muscle activity can be sensed by specific receptors (i.e., muscle spindles, golgi tendon organ), it is not under direct, conscious control. Through EMG feedback subjects are expected to develop an increased consciousness on how much their muscles contribute to a given task and thus to appropriately coordinate the activity of relevant muscles. The potentialities of EMG feedback for motor control and learning were first demonstrated when subjects learnt to control the recruitment and the discharge rate of motor units while observing and hearing motor unit action potentials (Basmajian, 1963). Since then, EMG feedback training has been extended to the general re-education of muscle activation in circumstances ranging from controlled, isometric contractions to functional, dynamic situations, in both healthy and pathological populations (e.g., Nouwen and Solinger, 1979; Poppen et al., 1988; Park and Yoo, 2012; Wright et al., 2013; Franz et al., 2014). Whether EMG feedback may be useful to improve posture control depends however on whether gaining a better control of muscle activation leads to a better control of posture.

Direct quantification of intrinsic ankle stiffness revealed insufficient values for the passive stabilization of standing posture (Loram and Lakie, 2002); neurally driven ankle torque is necessary to counterbalance gravity. How the nervous system actively modulates the ankle torque during standing is however a controversial issue (Morasso et al., 2014). Some suggest ankle torque is modulated continuously (Peterka, 2002; Masani et al., 2003). Others recently advanced the hypothesis that the control loop is open, with active adjustments in ankle torque being elicited intermittently via internal planning (Loram et al., 2009) or via center of mass position and velocity, threshold crossings (Bottaro et al., 2008). The current study does not presume one control mechanism—either continuous or intermittent, predominates over the other. Our rationale is based on the common observation (e.g., when learning movements) that improved, motor performance results from the suppression of unnecessary muscle activity (e.g., co-contraction). In addition to improve performance, and consistent with recent evidence (Bottaro et al., 2008; Kiemel et al., 2011), limiting muscle activation during standing leads to a more efficient control of posture without imposing major stabilization demands. Indeed, with EMG-biofeedback our participants were able to significantly repress muscle activation (Figure 4) without significantly increasing sway size (Figure 6). These observations are in agreement with theoretical and experimental findings arising, e.g., from stick balancing. Cabrera and Milton (2002) observed experimentally plausible angular displacements in stick balancing when the restoring force, applied to the stick by the hand, was set close to its lower, stability boundary in the parameter space. Most importantly, parametric noise associated with the restoring force resulted in corrective adjustments occurring at intervals both longer and shorter than the feedback delay; the stick is balanced occasionally (Cabrera and Milton, 2002). Results on human balancing in the frontal plane further substantiate the notion that more efficient, neural regulation of posture is achieved by tuning control parameters close to instability; that is, at wider stance widths, the muscular effort necessary for balancing decreases and so does the range of delayed, feedback gains (Bingham et al., 2011). Collectively, present and previous results suggest reducing muscle activity may be beneficial for the stable control of upright stance, providing the nervous system is capable of adapting to the situation.

Depending on the circumstances, reduction of muscle activation may not be the target parameter for EMG-biofeedback training. Parkinsonian subjects, for example, hardly coordinate muscle response and show exacerbated activation of ankle flexors to posterior translations of the supporting surface (Horak et al., 1992). EMG biofeedback, as implemented in the current study, could assist these subjects in minimizing synergists' activation. Subjects suffering from somatosensory loss, on the other hand, respond to postural, translational perturbations with markedly longer delays than controls (Inglis et al., 1994). For this specific population, using EMG biofeedback with the aim of minimizing muscle activity may be inappropriate; EMG-biofeeback could instead be implemented to assist these subjects in eliciting prompt muscle responses (e.g., to increase audio tone as quickly as possible in response to external perturbations or cues). Finally, and with the care of not excessively increasing the cognitive workload, EMG biofeedback could be integrated into existing feedback protocols to cover exigencies of a broad spectrum of inter-individual differences (Di Giulio et al., 2013; Morasso et al., 2014). More specifically, reducing muscle activity (EMG-based biofeedback) and reducing the amplitude of postural sways (Kinematic- and kinetic-based feedback) should therefore be conceived as complementary rather than competing approaches for the training of posture.

## Limitations and future perspectives

While current results suggest subjects can minimize muscle activity during standing when provided with EMG biofeedback, the lack of significant changes in CoP parameters must be interpreted carefully. Most importantly, CoP is not unequivocally related to body center of gravity (Bottaro et al., 2008; Kiemel et al., 2011). It is possible that with biofeedback subjects have learned to adopt a different posture, resulting in less muscle activation and no change in CoP mean position and displacement size. On one hand we were not able to quantify inter-segmental, kinematic changes in the present study. On the other hand, any inter-segmental changes that may have occurred in response to standing with EMG biofeedback were not large enough to be perceived by the experimenter. Moreover, as CoP reflects acceleration of the body center of mass, the lack of CoP difference in S3 and S4 conditions may indicate indeed an absence of changes in body motion. Even though ascertaining the existence and the relevance of postural changes with EMG biofeedback is not possible from current results, subjects have shown to be able to reduce muscular effort when provided with information otherwise not directly available to them; the degree of muscle activity.

Finally, some considerations on the generalization of current results are necessary. Poppen and colleagues (Poppen et al., 1988), for example, demonstrated that relaxation of trapezius muscle, achieved through EMG biofeedback, was retained in contexts other than that related to the feedback training. Even though EMG biofeedback is typically provided from a specific muscle, its effect manifests at different, synergist muscles (Wright et al., 2013; Franz et al., 2014). Whether the suppression of plantar flexors' activity observed here generalizes to other postural tasks, populations (e.g., elderly) and/or to other muscles is the subject of future investigations. It should be noted though that the lack of a significant decrease in lateral gastrocnemius activity during the feedback conditions (Figure 4) is not necessarily indicative of a lack of relaxation, it is probably accounted for by a floor effect; the amplitude of EMGs collected from this muscle during *standing at ease* is often within the background level (cf. Figure 3; see also Vieira et al., 2010; Héroux et al., 2014). As evidenced by current results, the well-documented benefits of EMG-biofeedback training may extend to the control of human standing posture.

## Author contributions

TV and AB conception and design of research. TV and AB analyzed data. TV, SB, and AB interpreted results of experiments. TV and AB prepared figures. TV, SB, and AB drafted manuscript. TV, SB, and AB edited and revised manuscript. TV, SB, and AB approved final version of manuscript.

## Funding

This study was supported by a national research project funded by the Italian Ministry of Education, Universities and Research (Protocol number: 2010R277FT), and co-funded by Compagnia di San Paolo and Fondazione C.R.T.

### Conflict of interest statement

The authors declare that the research was conducted in the absence of any commercial or financial relationships that could be construed as a potential conflict of interest.
