# Supplementary material for: Young, Healthy Subjects Can Reduce the Activity of Calf Muscles When Provided with EMG Biofeedback in Upright Stance
Source: Front Physiol. 2016 Apr 29;7:158. doi: 10.3389/fphys.2016.00158 (PMC4850153; doi:10.3389/fphys.2016.00158)
Supplement: Supplementary file 5 [file DataSheet1.DOCX]

Supplementary Material

Young, healthy subjects can minimise the activity of calf muscles when provided with EMG biofeedback in upright stance

Taian M. Vieira*, Stéphane Baudry, Alberto Botter

*** Correspondence:** Taian M. Vieira: taian.vieira@polito.it

# Supplementary Data

The supplementary material includes four movies. The image shown within each movie illustrates, from top to bottom, an example of the EMG envelope (thick, red trace) computed by low-pass filtering (5 Hz cut-off, 4th order Butterworth filter) the full-wave, rectified EMG (thin, green trace) and of the audio signals created by modulating a sinusoid from the EMG envelope according to the four different sensitivities considered in this study (see Methods); i.e., by setting the threshold *T_h_* to 30% (S1), 50% (S2), 70% (S3) and 90% (S4) of the 95^th^ percentile of the EMG envelope obtained during voluntary sways condition. The audio data of each of these movies was created using 8000 kS/s writing frequency and 8 bits resolution. Movie files were named according to the sensitivities considered to modulate the sinusoid from the EMG envelope (e.g., the vide file corresponding to the most sensitive condition, S1, was named as “Additional file 1_EMG-Audio_Standing_S1.avi”). For illustrative purpose, movies were created from the EMG collected from a single pair of electrodes when a participant stood upright naturally, without biofeedback.

**Name of supplementary videos:**

Additional file 1_EMG-Audio_Standing_S1

Additional file 2_EMG-Audio_Standing_S2

Additional file 3_EMG-Audio_Standing_S3

Additional file 4_EMG-Audio_Standing_S4

**Format of supplementary videos:**

All additional files are movies saved in AVI format
